# Secular trends and determinants of influenza vaccination uptake among patients with cardiovascular disease in Korea: Analysis using a nationwide database

**DOI:** 10.3389/fcvm.2022.961688

**Published:** 2022-10-04

**Authors:** Min Kim, Bumhee Yang, Seonhye Gu, Eung-Gook Kim, So Rae Kim, Kyeong Seok Oh, Woong-Su Yoon, Dae-Hwan Bae, Ju Hee Lee, Sang Min Kim, Woong Gil Choi, Jang-Whan Bae, Kyung-Kuk Hwang, Dong-Woon Kim, Myeong-Chan Cho, Hyun Lee, Dae-In Lee

**Affiliations:** ^1^Division of Cardiology, Department of Internal Medicine, Chungbuk National University Hospital, College of Medicine, Chungbuk National University, Cheongju, South Korea; ^2^Division of Pulmonary and Critical Care Medicine, Department of Internal Medicine, Chungbuk National University Hospital, College of Medicine, Chungbuk National University, Cheongju, South Korea; ^3^Department of Epidemiology and Health Informatics, Korea University, Seoul, South Korea; ^4^Department of Biochemistry, College of Medicine, Chungbuk National University, Cheongju, South Korea; ^5^Division of Pulmonary Medicine and Allergy, Department of Internal Medicine, Hanyang University College of Medicine, Seoul, South Korea

**Keywords:** influenza vaccination, cardiovascular disease, secular trend, risk factors, national immunization program

## Abstract

**Background:**

Influenza vaccination reduces cardiovascular events in patients with cardiovascular disease (CVD). Identifying the factors that affect influenza vaccination uptake can help improve the prognosis in patients with CVD. This study aimed to evaluate the secular trends of influenza vaccination uptake and factors associated with lack of vaccination in individuals with CVD.

**Materials and methods:**

We analyzed the annual trends and factors associated with influenza vaccination among 3,264 patients with CVD, included from the Korea National Health and Nutrition Examination Survey which reflect the health and nutritional status of the nationwide population of Korea conducted between 2007/2008 and 2018/2019. We used a stratified, multistage sampling method.

**Results:**

The influenza vaccination rate was greater in patients with CVD (53–74%) than in those without CVD (28–40%). Multivariable logistic regression analysis showed that age <50 years [odds ratio (OR), 16.22; 95% confidence interval (CI), 7.72–34.07], 50–64 years (OR, 6.71; 95% CI, 4.37–10.28), male sex (OR, 1.45; 95% CI, 1.14–1.65), and asthma (OR, 0.45; 95% CI, 0.22–0.92) were independently associated with a lack of influenza vaccination. Among patients aged <65 years, smoking (OR, 2.30; 95% CI, 1.31–4.04), college graduation status (OR, 1.81; 95% CI, 1.16–2.82), and hypertension (OR, 0.70; 95% CI, 0.51–0.95) were independently associated with influenza vaccination. For individuals aged 65years, there was no significant determinant of lack of vaccination.

**Conclusion:**

In patients with CVD, a continuous increase in the secular trend of influenza vaccination was demonstrated in Korea. Young age, male sex, and non-asthma status were independently associated with lack of influenza vaccination uptake.

## Highlights

### What is new?

- This cross-sectional survey explored the long-term secular trend and the factors influencing influenza vaccination uptake in patients with cardiovascular disease (CVD).
- We found the greater influenza vaccination rate in patients with CVD than their counterpart and these results were consistent in stratified analyses by age (<65 and 65 years) and sex (female and male).
- Age ≥65 years and asthma were positively associated, male sex was inversely associated with the influenza vaccination in patients with CVD.

### What are the clinical implications?

- Our findings highlight that a national immunization program (NIP) for high-risk population can attribute to higher influenza vaccination rate and attenuate the health, behavioral, and sociodemographic disparities.

## Introduction

Cardiovascular disease (CVD) is the leading cause of morbidity and mortality globally (1). According to the World Health Organization (WHO), approximately 17.9 million people died from CVD in 2019, accounting for 32% of the global deaths (2). Respiratory viral infections, such as influenza are known to increase the risk of development of CVD (3). It is also known that underlying CVD is associated with an increased risk of complications from influenza, including increased morbidity, mortality, and hospitalization (4, 5). Therefore, it is important to prevent influenza in patients with CVD.

In individuals with CVD, influenza vaccination significantly reduced all-cause mortality, recurrence of CVD, cardiovascular death, and hospitalizations for heart failure (6). Therefore, influenza vaccines are considered essential in CVD management and prevention. Considering that influenza is vaccine-preventable, international guidelines recommend that high-risk populations with CVD receive an influenza vaccine every year (7, 8), and the incorporation of vaccination into routine patient care for CVD prevention requires a paradigm change in clinical practice.

However, despite the importance of influenza vaccination in patients with CVD, few studies have investigated the coverage of influenza vaccination and the factors that affect vaccination in such patients (9). Therefore, we aimed to investigate the secular trend of influenza vaccine uptake and factors associated with the lack of vaccination in individuals with CVD, using a recent nationwide observational study.

## Materials and methods

### Study population

The Korea National Health and Nutrition Examination Survey (NHANES) is a population-based nationwide survey conducted by the Korea Disease Control and Prevention Agency (KCDC) to assess the health and nutritional status of the non-institutionalized population of Korea. We used data from NHANES IV (2007–2009), V (2010–2012), VI (2014–2015), VII (2016–2018), and VIII (2019). Data from NHANES V (2013) were excluded because vaccination data were not available. The study population was investigated using a stratified, multistage sampling method.

**FIGURE 1:**
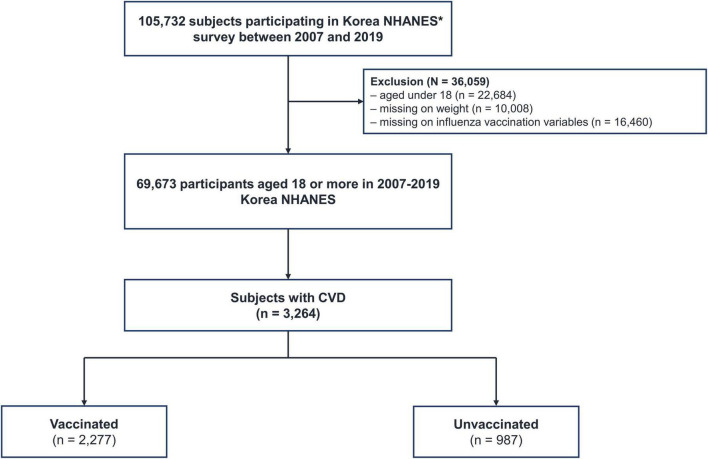
Flow chart of the study population. CVD, cardiovascular disease; NHANES, National Health and Nutrition Examination Survey. *Data from NHANES V (2013) were excluded because vaccination data were not available.

Between 2007 and 2019, there were 1,05,732 participants, of which 22,684 aged <18 years, 10,008 with missing weighted variables (*n* = 10,008), and 16,460 with missing survey data on influenza vaccination coverage were excluded; thus, 69,673 subjects were included. Among them, 3,264 (9.7%) had CVD and were classified into vaccinated (*n* = 2,277) and unvaccinated (*n* = 987) groups (Figure 1). The study protocol was approved by the institutional review board of the Chungbuk National University Hospital (application no. 2022-05-002).

### Exposure: Cardiovascular disease

The main exposure in this study was CVD, which was defined as a self-reported physician’s diagnosis of any of the following: stroke, myocardial infarction, or angina (9).

### Outcomes: Influenza vaccination

The main outcomes of our study were the trend of influenza vaccination and the factors associated with influenza vaccination in patients with CVD. Influenza vaccination was investigated using the following question: “Have you ever been vaccinated against influenza in the past year?”

### Confounding variables

Data on age, sex, body mass index (BMI), smoking history, alcohol consumption, marital status, income, area of residence, education, and cardiac symptoms were obtained from the NHANES database. Comorbidities of stroke, asthma, pulmonary tuberculosis, and chronic kidney disease were self-reported based on previous physician diagnoses (10–13). Chronic obstructive pulmonary disease (COPD) was defined based on pre-bronchodilator forced expiratory volume in 1 s (FEV_1_)/forced vital capacity (FVC) < 0.7 (14). Diabetes mellitus (DM) was defined as the presence of any of the following: (1) history of diagnosis of DM by a physician, (2) fasting glucose level ≥ 126 mg/dl, (3) hemoglobin A1c (HbA1c) ≥ 6.5% (15), or (4) intake of oral hypoglycemic agents or insulin (16). Hypertension was defined as follows: a self-reported physician diagnosis, the use of antihypertensive medication, systolic blood pressure ≥ 140 mmHg, or diastolic blood pressure ≥ 90 mmHg (13, 17). Dyslipidemia was defined through a self-reported physician diagnosis, the use of lipid-lowering medication, a total cholesterol level ≥ 240 mg/dl, or a fasting triglyceride level ≥ 200 mg/dl (10, 13). Marital status was classified into three groups (unmarried, married, widowed/separated/divorced) and household income level was classified into four groups by quartile division (9). Drinking behavior was divided into two groups (non-heavy drinker, heavy drinker) by using the Alcohol Use Disorders Identification Test (≥12 scores: heavy, <12 scores: non-heavy) (18).

### Statistical analysis

All analyses were performed using survey commands in STATA (version 15.1; StataCorp LP, College Station, TX, USA) to account for complex sampling design and survey weights. All data are presented as weighted percentages with standard errors (SE). The data were compared using Student’s t-test for continuous variables and Pearson’s χ2 test for categorical variables. Multivariate analyses were performed by adjusting for significant variables in the univariate logistic regression analyses. All tests were two-sided, and statistical significance was set at *p* < 0.05.

## Results

### Baseline characteristics

In this study, 3,264 (3.1%) of 1,05,732 individuals had CVD, of which 1,871 had coronary artery disease (CAD) (1.8%) and 1,393 (1.3%) had ischemic stroke (Figure 1). The baseline characteristics of vaccinated and unvaccinated individuals with CVD are summarized in Table 1. Nearly one-third of patients with CVD were not vaccinated against influenza. A lack of influenza vaccine uptake was significantly higher among younger individuals, males (65.4 vs. 50.5%, *p* < 0.001), those who smoke (28.3 vs. 13.1%, *p* < 0.001) and drink heavily (15.7 vs. 6.8%, *p* < 0.001), households with high income (23.2 vs. 12.3%, *p* < 0.001), those who live in urban areas (79.3 vs. 75.5%, *p* = 0.024), unmarried status (5.2 vs. 1.6%, *p* < 0.001), and college graduates (20.4 vs. 8.8%, *p* < 0.001). In contrast, the influenza vaccine uptake rate was higher in individuals with comorbidities, including asthma (6.2 vs. 4.1%, *p* = 0.045), DM (35.2 vs. 28.9%, *p* = 0.005), hypertension (71.1 vs. 57.8%, *p* < 0.001), and chronic kidney disease (18.1 vs. 10.1%, *p* < 0.001) than in individuals without comorbidities.

**TABLE 1:** Baseline characteristics of the study population.

	Total (*N* = 3,264)	Vaccinated (*N* = 2,277)	Unvaccinated (*N* = 987)	*P*-value
**Study population**				
Angina or MI	57.3 (1.1)	57.5 (1.3)	57.0 (1.9)	0.837
Stroke	47.5 (1.1)	47.9 (1.3)	46.6 (2.0)	0.568
**Age, years**	65.4 (0.3)	69.1 (0.3)	58.6 (0.4)	<0.001
**Sex, male**	55.7 (1.0)	50.5 (1.2)	65.4 (1.8)	<0.001
**BMI, kg/m^2^**				0.221
Underweight (<18.5)	2.0 (0.3)	1.7 (0.3)	2.4 (0.6)	
Normal weight (18.5–24.9)	55.8 (1.0)	57.0 (1.2)	53.7 (1.9)	
Overweight/obesity (≥25)	42.3 (1.1)	41.3 (1.2)	44.0 (1.9)	
**Smoking status**				<0.001
Non-smoker	46.5 (1.1)	51.8 (1.2)	36.7 (1.8)	
Past smoker	35.1 (1.1)	35.2 (1.2)	35.0 (1.9)	
Current smoker	18.4 (0.9)	13.1 (0.9)	28.3 (1.9)	
**Alcohol consumption**				<0.001
Non-heavy drinker	90.1 (0.7)	93.2 (0.7)	84.3 (1.5)	
Heavy drinker	9.9 (0.7)	6.8 (0.7)	15.7 (1.5)	
**Marital status**				<0.001
Unmarried	2.9 (0.4)	1.6 (0.4)	5.2 (1.0)	
Married	72.9 (1.0)	70.7 (1.2)	77.0 (1.6)	
Widowed/Separated/Divorced	24.2 (0.9)	27.7 (1.2)	17.8 (1.4)	
**Income**				<0.001
Low	38.4 (1.1)	44.3 (1.3)	27.4 (1.7)	
Intermediate	45.5 (1.1)	43.4 (1.3)	49.3 (2.0)	
High	16.2 (0.9)	12.3 (0.9)	23.3 (1.8)	
**Area of residence**				0.024
Urban	76.9 (1.2)	75.5 (1.3)	79.3 (1.6)	
Rural	23.1 (1.2)	24.5 (1.3)	20.7 (1.6)	
**Education**				<0.001
Elementary school graduate	46.8 (1.1)	53.8 (1.3)	34.1 (1.8)	
Middle/High school graduate	40.3 (1.0)	37.5 (1.2)	45.5 (1.9)	
College graduate	12.9 (0.8)	8.8 (0.8)	20.4 (1.6)	
**Symptoms**				
Chest pain	6.4 (0.9)	5.7 (1.0)	7.4 (1.5)	0.298
Dyspnea	3.9 (0.7)	3.3 (0.7)	4.8 (1.4)	0.304
Palpitation	5.5 (0.5)	6.1 (0.6)	4.5 (0.8)	0.120
**Comorbidities**				
COPD*	20.1 (1.1)	22.2 (1.4)	16.6 (1.8)	0.014
Asthma	5.4 (0.5)	6.2 (0.6)	4.1 (0.7)	0.045
Pulmonary TB	6.0 (0.5)	6.6 (0.6)	4.9 (0.8)	0.113
DM	33.0 (1.0)	35.2 (1.2)	28.9 (1.8)	0.005
Hypertension	66.4 (1.0)	71.1 (1.2)	57.8 (1.9)	<0.001
Dyslipidemia	69.9 (1.0)	69.56 (1.14)	70.4 (1.8)	0.672
Chronic kidney disease	15.6 (0.8)	18.53 (0.98)	10.1 (1.1)	<0.001

*COPD was defined based on a pre-bronchodilator FEV1/FVC < 0.7.

BMI, body mass index; COPD, chronic obstructive pulmonary disease; DM, diabetes mellitus; FEV1/FVC, forced expiratory volume in 1 s/forced vital capacity; MI, myocardial infarction; TB, tuberculosis.

### Secular trends of influenza vaccination uptake

**FIGURE 2:**
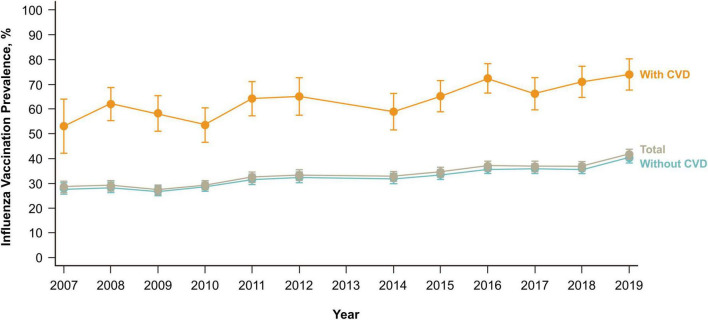
Influenza vaccination trends over 9 years according to the cardiovascular disease history. CVD, cardiovascular disease.

**FIGURE 3:**
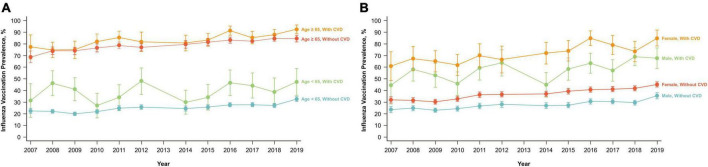
Influenza vaccination trends over 9 years according to the cardiovascular disease history. **(A)** Age (<65 and ≥65 years). **(B)** Sex (male and female). CVD, cardiovascular disease.

The vaccine uptake rate in individuals with CVD was significantly higher than that in individuals without CVD (*p* < 0.01). Regardless of the presence of CVD, influenza vaccination coverage displayed an upward trend from 2007/2008 to 2018/2019 (Figure 2). The influenza vaccination uptake rate in individuals with CVD increased from 53% in 2007/2008 to 74% in 2018/2019. However, in individuals without CVD, the influenza vaccination coverage rate was 40% in 2019. Similar trends were observed in stratified analyses by age group and sex (Figure 3). Within the CVD category (i.e., among individuals with or without CVD), individuals aged ≥65 years had a higher vaccination rate than individuals aged <65 years, and females had a higher vaccine uptake rate than males. The influenza vaccine uptake rate of individuals with CVD during the 2018/2019 season reached 91.4% for individuals aged ≥65 years but was less than 47.3% for individuals aged <65 years.

### Predictor of the lack of influenza vaccination uptake

Multivariable logistic analysis (Table 2) showed that the clinically independent factor with the highest odds of lack of influenza vaccination was age <50 years [odds ratio (OR), 16.22; 95% confidence interval (CI), 7.72–34.07], followed by age of 50–64 years (OR, 6.71; 95% CI, 4.37–10.28), and male sex (OR, 1.45; 95% CI, 1.14–1.65). In contrast, asthma was independently and positively associated with influenza vaccination (for the lack of vaccination OR = 0.45; 95% CI, 0.22–0.92).

**TABLE 2:** Factors associated with influenza unvaccinated status in patients with cardiovascular disease.

	Univariable		Multivariable	
		
	OR (95% CI)	*P*-value	OR (95% CI)	*P*-value
**Age**				
<50	16.69 (10.98, 25.36)	<0.001	16.22 (7.72, 34.07)	<0.001
50–65	7.24 (5.92, 8.86)	<0.001	6.71 (4.37, 10.28)	<0.001
≥65	Reference		Reference	
**Sex, male**	1.46 (1.35, 1.55)	<0.001	1.45 (1.14, 1.65)	0.009
**BMI, kg/m^2^**				
Underweight (<18.5)	1.47 (0.81, 2.69)	0.207	–	
Normal weight (18.5–24.9)	Reference			
Overweight/obesity (≥25)	1.13 (0.94, 1.35)	0.184	–	
**Smoking status**				
Non-smoker	Reference		Reference	
Past smoker	1.40 (1.14, 1.72)	0.001	0.86 (0.55, 1.33)	0.489
Current smoker	3.05 (2.39, 3.91)	<0.001	1.44 (0.88, 2.36)	0.148
**Alcohol consumption**				
Non-heavy drinker	Reference		Reference	
Heavy drinker	2.55 (1.88, 3.46)	<0.001	1.28 (0.80, 2.05)	0.296
**Marital status**				
Unmarried	Reference		Reference	
Married	0.34 (0.19, 0.62)	<0.001	1.18 (0.37, 3.79)	0.778
Widowed/Separated/Divorced	0.20 (0.11, 0.38)	<0.001	1.17 (0.35, 3.95)	0.797
**Income**				
Low	0.33 (0.25, 0.43)	<0.001	0.84 (0.52, 1.35)	0.464
Intermediate	0.60 (0.46, 0.78)	<0.001	0.91 (0.61, 1.38)	0.669
High	Reference		Reference	
**Area of residence**				
Urban	1.24 (1.03, 1.50)	0.024	1.11 (0.80, 1.55)	0.520
Rural	Reference		Reference	
**Education**				
Elementary school graduate	Reference		Reference	
Middle/High school graduate	1.92 (1.57, 2.34)	<0.001	0.85 (0.61, 1.19)	0.341
College graduate	3.67 (2.75, 4.88)	<0.001	1.16 (0.72, 1.86)	0.549
**Symptoms**				
Chest pain	1.33 (0.77, 2.29)	0.300	–	
Dyspnea	1.46 (0.71, 3.00)	0.307	–	
Palpitation	0.72 (0.48, 1.09)	0.121	–	
**Comorbidities**				
COPD	0.69 (0.52, 0.93)	0.015	1.38 (0.96, 1.98)	0.084
Asthma	0.65 (0.43, 0.99)	0.046	0.45 (0.22, 0.92)	0.029
Pulmonary TB	0.73 (0.49, 1.08)	0.114	–	
DM	0.75 (0.62, 0.92)	0.005	0.85 (0.60, 1.20)	0.358
Hypertension	0.56 (0.46, 0.67)	<0.001	0.94 (0.69, 1.28)	0.700
Dyslipidemia	1.04 (0.86, 1.27)	0.672	–	
Chronic kidney disease	0.50 (0.38, 0.65)	<0.001	0.77 (0.51, 1.17)	0.222

BMI, body mass index; COPD, chronic obstructive pulmonary disease; DM, diabetes mellitus; MI, myocardial infarction; TB, tuberculosis.

Tables 3, 4 show multivariate logistic analyses after stratification for age (<65 and ≥65 years). Individuals <65 years, current smokers (OR, 2.30; 95% CI, 1.31–4.04), and college graduates (OR, 1.81, 95% CI, 1.16–2.82) were significant independent predictors for the lack of influenza vaccination uptake, but hypertension was positively associated with influenza vaccination (OR, 0.70, 95% CI, 0.51–0.95). For individuals aged ≥65 years, there was no significant determinant of lack of vaccination uptake.

**TABLE 3:** Factors associated with influenza unvaccinated status in patients aged <65 years with cardiovascular disease.

	Univariable		Multivariable	
		
	OR (95% CI)	*P*-value	OR (95% CI)	*P*-value
**Sex, male**	1.41 (1.20, 1.56)	0.001	1.10 (0.48, 1.56)	0.691
**BMI, kg/m^2^**				
Underweight (<18.5)	0.98 (0.34, 2.82)	0.973	–	
Normal weight (18.5–24.9)	Reference			
Overweight/obesity (≥25)	1.07 (0.81, 1.42)	0.630	–	
**Smoking status**				
Non-smoker	Reference		Reference	
Past smoker	1.38 (0.99, 1.92)	0.055	1.22 (0.71, 2.10)	0.471
Current smoker	2.44 (1.66, 3.59)	0.000	2.30 (1.31, 4.04)	0.004
**Alcohol consumption**				
Non-heavy drinker	Reference			
Heavy drinker	1.40 (0.93, 2.10)	0.103	–	
**Marital status**				
Unmarried	Reference			
Married	0.72 (0.37, 1.43)	0.347	–	
Widowed/Separated/Divorced	0.59 (0.28, 1.24)	0.162	–	
**Income**				
Low	0.69 (0.46, 1.04)	0.074	–	
Intermediate	0.82 (0.57, 1.16)	0.259	–	
High	Reference			
**Area of residence**				
Urban	1.20 (0.88, 1.62)	0.250	–	
Rural	Reference			
**Education**				
Elementary school graduate	Reference		Reference	
Middle/High school graduate	1.28 (0.93, 1.76)	0.135	1.10 (0.78, 1.54)	0.586
College graduate	2.14 (1.40, 3.27)	0.000	1.81 (1.16, 2.82)	0.009
**Symptoms**				
Chest pain	1.42 (0.69, 2.96)	0.343	–	
Dyspnea	2.12 (0.76, 5.96)	0.152	–	
Palpitation	0.80 (0.40, 1.62)	0.541	–	
**Comorbidities**				
Asthma	0.57 (0.29, 1.12)	0.105	–	
Pulmonary TB	0.61 (0.32, 1.16)	0.133	–	
DM	0.76 (0.55, 1.05)	0.091	–	
Hypertension	0.71 (0.53, 0.95)	0.022	0.70 (0.51, 0.95)	0.022
Dyslipidemia	1.00 (0.73, 1.37)	0.991	–	
Chronic kidney disease	0.71 (0.45, 1.12)	0.141	–	

BMI, body mass index; COPD, chronic obstructive pulmonary disease; DM, diabetes mellitus; MI, myocardial infarction; TB, tuberculosis.

**TABLE 4:** Factors associated with influenza unvaccinated status in patients aged ≥65 years with cardiovascular disease.

	Univariable		Multivariable	
		
	OR (95% CI)	*P*-value	OR (95% CI)	*P*-value
**Sex, male**	1.24 (1.01, 1.42)	0.040	1.20 (0.75, 1.48)	0.333
**BMI, kg/m^2^**				
Underweight (<18.5)	2.40 (1.05, 5.48)	0.038	–	
Normal weight (18.5–24.9)	Reference			
Overweight/obesity (≥25)	0.96 (0.74, 1.26)	0.787	–	
**Smoking status**				
Non-smoker	Reference		Reference	
Past smoker	1.16 (0.87, 1.56)	0.316	0.97 (0.62, 1.52)	0.903
Current smoker	1.70 (1.15, 2.51)	0.008	1.63 (0.99, 2.69)	0.057
**Alcohol consumption**				
Non-heavy drinker	Reference			
Heavy drinker	1.69 (0.98, 2.92)	0.060	–	
**Marital status**				
Unmarried	Reference			
Married	1.71 (0.29, 10.30)	0.556	–	
Widowed/Separated/Divorced	2.01 (0.34, 12.07)	0.443	–	
**Income**				
Low	0.75 (0.48, 1.16)	0.198	–	
Intermediate	0.78 (0.50, 1.21)	0.267	–	
High	Reference			
**Area of residence**				
Urban	0.98 (0.75, 1.28)	0.863	–	
Rural	Reference			
**Education**				
Elementary school graduate	Reference			
Middle/High school graduate	0.80 (0.60, 1.08)	0.146	–	
College graduate	1.18 (0.71, 1.94)	0.524	–	
**Symptoms**				
Chest pain	0.91 (0.36, 2.34)	0.850	–	
Dyspnea	1.13 (0.39, 3.31)	0.825	–	
Palpitation	1.13 (0.65, 1.96)	0.656	–	
**Comorbidities**				
Asthma	1.03 (0.60, 1.78)	0.906	–	
Pulmonary TB	0.95 (0.59, 1.54)	0.841	–	
DM	0.82 (0.63, 1.09)	0.169	–	
Hypertension	0.91 (0.67, 1.24)	0.565	–	
Dyslipidemia	0.96 (0.72, 1.26)	0.749	–	
Chronic kidney disease	0.75 (0.53, 1.06)	0.102	–	

BMI, body mass index; COPD, chronic obstructive pulmonary disease; DM, diabetes mellitus; MI, myocardial infarction; TB, tuberculosis.

## Discussion

We examined the secular trend and determinants of influenza vaccine uptake in individuals in South Korea. Overall, the vaccination uptake rate in individuals with CVD increased by 21% during 12 seasons (2007/2008 to 2018/2019), reaching 74% in the 2018/2019 season. Although the influenza vaccine uptake rate in individuals with CVD was suboptimal considering the goal of 90% in high-risk conditions, proposed by the Healthy People 2020 initiative of the United States (US) (19), it almost meets the vaccine uptake goal of 75% proposed by the European Union (EU) Council (20). Moreover, this proportion in South Korea was higher than that of chronically ill individuals in the EU (ranging from about 20% in Norway to 60% in Northern Ireland) (20) and the US (67%) (21).

However, in this study, the vaccination rate in patients aged <65 years was only 47%. The influenza under-vaccination trend in younger individuals has been well-elucidated in many studies (22–24), with older individuals being more likely to visit clinics, have chronically ill status, and frequently recommended for vaccination uptake by health care providers (25). However, the relative odds of those aged 18–49 years (OR, 16.22) and 50–64 years (OR, 6.71) for lacking vaccine uptake was three to seven times higher than that of individuals with CVD aged 40–64 years in the US (OR, 2.32; 95% CI 2.06–2.62) (26). The prominently higher influenza vaccination uptake in CVD patients aged ≥65 years in South Korea can be attributed to a substantial national immunization program (NIP) for those aged ≥65 years, which was continuously developed for two decades (27), and not merely by the effect of age as a sociodemographic factor.

As influenza vaccination was provided free of charge to all individuals aged ≥65 years in 2005, the influenza vaccination rate among individuals aged ≥65 years gradually increased from 40% in 2005 to 60% in 2009. However, during the H1N1 pandemic of 2009, the government of South Korea had difficulty in meeting the sudden increase in vaccine demand and thereafter ramped up the domestic production system to meet 70% of the vaccine requirement. This production and supply innovation in influenza vaccination has resulted in a second increase in the influenza vaccine uptake rate by improving access to vaccination. Recently, the expansion of the influenza NIP to primary care clinics has enhanced the influenza vaccination rates to 82.4% in individuals aged ≥65 years, in the 2016–2017 season. Moreover, as indicated by the multivariable analysis in this study, the series of NIP administered to all individuals aged ≥65 years can reduce the effect of disparity in health, behavioral, and sociodemographic factors on influenza vaccination uptake.

A 25% reduction in all-cause mortality of CAD patients by influenza vaccination was comparable to the effect of beta-blockage and angiotensin-converting enzyme inhibitors on mortality reduction of 20–25% (28). In a subgroup analysis of a recent intervention trial, influenza vaccination in patients with acute myocardial infarction was beneficial in preventing potential serious complications including all-cause death, myocardial infarction, or stent thrombosis regardless of age (29). Therefore expanding the free coverage of vaccination to CVD individuals aged <65 years may be a robust measure to attenuate the complication of influenza infection in individuals with CVD. However, studies on the cost-effectiveness of influenza vaccination have been limited to children, pregnant women, and the elderly (30), and have not focused on individuals with CVD aged <65 years (31).

Recently, a remarkable study comparing the effect of influenza vaccination to placebo administered shortly after acute coronary syndrome demonstrated the considerable risk reduction in all-cause mortality and cardiovascular death without increased adverse effect of influenza vaccination (29). In this regard, as same manner of prescribing antiplatelets or statins, it can be considered to add the clinical routine practice of checking whether CVD patients were vaccinated against influenza before discharge and recommending vaccination uptake during hospitalization. Moreover, the guideline revision to place the priority of influenza on an equal position with pharmacotherapy including antiplatelets and statins for secondary prevention in CVD patients and policy interventions to support influenza vaccination before discharge can contribute to improving influenza vaccination uptake rate and cardiovascular mortality. However, given the meta-analysis that statins mitigate short-term (30 days) and mid-term mortality (90 days) in patients infected with influenza by their pleotropic effect mediating anti-inflammation and immunomodulation (32), it should not be underestimated that other pharmacotherapy including statins are also important in ameliorating the health effects of influenza in CVD patients.

As the influenza vaccination uptake rate was greatly influenced by age group, we analyzed the determinants of the lack of influenza vaccination according to the subgroup stratified by the cutoff value of 65 years. This study showed that individuals with CVD aged <65 years with current smoking habits were likely to lack influenza vaccination, which is in line with the results of previous studies based on the Korea NHANES database (33–35) and studies conducted in the US (36), United Kingdom (37), and Italy (38). This can be explained by positive health-seeking behaviors and motivation in individuals with smoking cessation or negative health-seeking behaviors in current smokers (39). Smoking-induced disruption of respiratory epithelial cell lining increases susceptibility to influenza (40), which leads to a higher risk of hospital or intensive care unit admission after influenza infection (41). The effect of smoking on CVD recurrence can be exacerbated by inflammatory release of cytokines, thrombogenic status, hypoxia, and tachycardia after influenza infection (42). Therefore, a government campaign or healthcare provider’s direct recommendation to motivate the influenza vaccine uptake tailored for current smokers is needed to prevent the harmful interaction of influenza, smoking, and CVD.

In this study, the educational status of college graduates was another determinant of the lack of influenza vaccination in CVD individuals aged <65 years. Moreover, a paradoxical statistical trend was observed between low household income and higher vaccination rates. These findings can be attributed to the financial reimbursement for influenza vaccination provided by local municipalities to the lower quartile income bracket of individuals aged 50–64 years (43). On the contrary, the decreased opportunity for influenza vaccination associated with lower educational level was identified in Austria and Poland where there is no public reimbursement for influenza vaccination (23). A recent study in the US similarly showed that lower education levels and lower household incomes were related to a lack of vaccination (26). These observations suggest that socioeconomic factors, including education and income level, are modifiable variables that depend on the degree of public reimbursement, and are not an independent determinant for lack of influenza uptake in individuals with CVD.

The lower rates of influenza vaccination in men as seen in this study has been observed in previous studies conducted in Korea (33, 44, 45). Although the reasons for this phenomenon are being speculated, a probable cause could be unhealthy lifestyle in men. It is also possible that women comply more than men with clinicians’ recommendations for influenza vaccination. Supporting this hypothesis, a previous study in Korea showed that medication adherence was higher in women than in men (46).

Among the comorbidities, asthma had a significant influence on vaccination in individuals with CVD. Several studies have demonstrated the effectiveness of vaccination in patients with asthma. Vaccination against influenza prevented nearly half of influenza infections and about 60–80% of asthma attacks (47, 48). Therefore, the current guidelines recommend that subjects with asthma should be vaccinated against the influenza virus every year (49–51). As a result, a study using national data from Korea reported that the influenza vaccination rate of subjects with asthma was significantly higher than that of the general population. The vaccination rate increases with age; in particular, 81.5% of asthmatic patients aged >65 years receive the influenza vaccine (52, 53).

This study had several limitations. First, several variables were not included in the NHANES database, such as the perception of benefits and adverse effects of influenza vaccine, access level to vaccine, provider recommendation, and exposure to media, and were therefore not evaluated. Second, the study based on the NHANES database can imply the possibility of recall bias, as there was a gap between the survey period conducted year-round and the vaccination period from September to December. Third, the causal relationship between the lack of vaccination and variables could not be determined due to the cross-sectional design of this study. Forth, we acknowledge that this study did not demonstrate whether policy implemented by Korean government directly improved mortality in patients with CVD. However, this study has strength in that it showed a secular trend of influenza vaccination during long term follow-up, and it analyzed more detailed individual-level factors in patients with CVD based on a nationwide survey statistically reflecting the entire population of South Korea compared to previous similar study (9). Moreover, this study demonstrated an example that comprehensive and long-term measures implemented by national interventions could increase the coverage of influenza vaccination uptake.

## Conclusion

In conclusion, this study showed a continually improving secular trend in influenza vaccine uptake among individuals with CVD in South Korea. This can be attributed to the organized national influenza vaccination program, which underscores public support for influenza vaccination and reduces socioeconomic disparity at the individual level among high-risk individuals vulnerable to influenza infection. However, it is still necessary to develop measures for improving influenza vaccine uptake in individuals with CVD aged <65 years.

## Data availability statement

The raw data supporting the conclusions of this article will be made available by the authors, without undue reservation.

## Ethics statement

The studies involving human participants were reviewed and approved by the institutional review board of the Chungbuk National University Hospital. Written informed consent for participation was not required for this study in accordance with the national legislation and the institutional requirements.

## Author contributions

D-IL, HL, BY, and MK: conceptualization, validation, and writing—original draft, review, and editing. D-IL, HL, BY, SG, and MK: data curation, formal analysis, and software. D-IL, HL, BY, E-GK, SRK, KO, W-SY, D-HB, JL, SMK, SG, J-WB, K-KH, D-WK, M-CC, and MK: methodology and investigation. All authors contributed to the article and approved the submitted version.

## Abbreviations

CVD: cardiovascular disease
EU: European Union
KCDC: Korea Disease Control and Prevention Agency
KNHANES: Korea National Health and Nutrition Examination Survey
NIP: national immunization program
US: United States
WHO: World Health Organization.
